# The Effectiveness of Serious Games for Alleviating Depression: Systematic Review and Meta-analysis

**DOI:** 10.2196/32331

**Published:** 2022-01-14

**Authors:** Alaa Abd-Alrazaq, Eiman Al-Jafar, Mohannad Alajlani, Carla Toro, Dari Alhuwail, Arfan Ahmed, Shuja Mohd Reagu, Najeeb Al-Shorbaji, Mowafa Househ

**Affiliations:** 1 Division of Information and Computing Technology College of Science and Engineering Hamad Bin Khalifa University, Qatar Foundation Doha Qatar; 2 Health Informatics & Information Management Department Faculty of Allied Health Sciences Kuwait University Kuwait Kuwait; 3 Institute of Digital Healthcare Warwick Manufacturing Group University of Warwick Warwick United Kingdom; 4 Information Science Department Kuwait University Kuwait Kuwait; 5 Health Informatics Unit Dasman Diabetes Institute Kuwait Kuwait; 6 AI Center for Precision Health Weill Cornell Medicine-Qatar Doha Qatar; 7 Mental Health Services Hamad Medical Corporation Doha Qatar; 8 eHealth Development Association of Jordan Amman Jordan

**Keywords:** serious games, exergames, depression, cognitive behavioral therapy, systematic reviews, meta-analysis

## Abstract

**Background:**

Depression is a common mental disorder characterized by disturbances in mood, thoughts, or behaviors. Serious games, which are games that have a purpose other than entertainment, have been used as a nonpharmacological therapeutic intervention for depression. Previous systematic reviews have summarized evidence of effectiveness of serious games in reducing depression symptoms; however, they are limited by design and methodological shortcomings.

**Objective:**

This study aimed to assess the effectiveness of serious games in alleviating depression by summarizing and pooling the results of previous studies.

**Methods:**

A systematic review of randomized controlled trials (RCTs) was conducted in accordance with the PRISMA (Preferred Reporting Items for Systematic Reviews and Meta-Analyses) statement. The search sources included 6 bibliographic databases (eg, MEDLINE, PsycINFO, IEEE Xplore), the search engine “Google Scholar,” and backward and forward reference list checking of the included studies and relevant reviews. Two reviewers independently carried out the study selection, data extraction, risk of bias assessment, and quality of evidence appraisal. Results of the included studies were synthesized narratively and statistically, as appropriate, according to the type of serious games (ie, exergames or computerized cognitive behavioral therapy [CBT] games).

**Results:**

From an initial 966 citations retrieved, 27 studies met the eligibility criteria, and 16 studies were eventually included in meta-analyses. Very low-quality evidence from 7 RCTs showed no statistically significant effect of exergames on the severity of depressive symptoms as compared with conventional exercises (*P*=.12). Very low-quality evidence from 5 RCTs showed a statistically and clinically significant difference in the severity of depressive symptoms (*P*=.004) between exergame and control groups, favoring exergames over no intervention. Very low-quality evidence from 7 RCTs showed a statistically and clinically significant effect of computerized CBT games on the severity of depressive symptoms in comparison with no intervention (*P*=.003).

**Conclusions:**

Serious games have the potential to alleviate depression as other active interventions do. However, we could not draw definitive conclusions regarding the effectiveness of serious games due to the high risk of bias in the individual studies examined and the low quality of meta-analyzed evidence. Therefore, we recommend that health care providers consider offering serious games as an adjunct to existing interventions until further, more robust evidence is available. Future studies should assess the effectiveness of serious games that are designed specifically to alleviate depression and deliver other therapeutic modalities, recruit participants with depression, and avoid biases by following recommended guidelines for conducting and reporting RCTs.

**Trial Registration:**

PROSPERO International Prospective Register of Systematic Reviews CRD42021232969; https://www.crd.york.ac.uk/prospero/display_record.php?RecordID=232969

## Introduction

### Background

An individual's mental health is fundamental to living a healthy and enjoyable lifestyle. Studies estimate that 1 in 3 people may suffer from a mental illness during their lifetime [1]. The World Health Organization (WHO) reports that depression is a “leading cause of disability worldwide and is a major contributor to the overall global burden of disease” affecting more than 264 million people of all ages globally [2]. Depression is a mental health disorder that the Sustainable Development Goals of the United Nations (2015) has listed among its 270 targets and 230 indicators. Depressive disorders account for most of the total disability-adjusted life years globally. Although depressive disorders are global, they particularly affect those living in high and upper-middle-income nations [3]. This heavy toll is exacerbated by the fact that up to one-half (50%) of the people living in high-income countries and 90% of those living in low-resource settings receive no treatment for depressive disorders [4].

Depressive disorders are a family of mental disorders ranging in severity from mild temporary episodes of sadness to more severe and persistent depression [5]. Depressive disorders include disruptive mood dysregulation disorder, premenstrual dysphoric disorder, substance- or medication-induced depressive disorder, and major depressive disorder [6]. Depressive disorders are characterized by disturbances in mood, thoughts, or behaviors. Furthermore, depressive disorders do not affect the mind alone but are reported to impact a person’s body [7,8]. Depression is known to be caused by a number of factors that interact in complex mechanisms: social, genetic, pathological, and chemical [9]. Treatments for depressive disorders are generally classified into either pharmacological or psychosocial (ie, nonpharmacological) interventions. Pharmacological treatments involve the use of drugs (eg, antidepressants) while examples of psychosocial treatments include cognitive behavioral therapy (CBT), exposure therapy, and exercise [10].

The use of serious games, defined as games that have a purpose other than entertainment, has seen a rise in recent years [11]. Serious games use elements unique to gaming in order to educate or influence change in experience or behaviors [12]. Several industries have adopted and continue to use serious games including health care, education, and airlines [13]. Among other things, serious games have been effectively utilized for education, prevention, and treatment of chronic conditions (eg, asthma and diabetes) [14], therapeutic rehabilitation [15], and educational resources for health care professionals [16]. Moreover, serious games have been used as a nonpharmacological therapeutic intervention for mental disorders [17]. Serious games have been utilized as a treatment for various mental disorders, including depression [18,19], anxiety [20], posttraumatic stress disorder [21,22], autism spectrum disorder [23,24], dementia [25,26], alcohol use disorder [27], attention deficit hyperactivity disorder [28], and obsessive-compulsive disorder [13,29].

Gaming as a therapeutic tool in mental health can potentially offer several specific advantages that may be missing from traditional forms of delivery. The gaming industry is, as ever, popular globally [30] and arguably easier to access than even basic mental health services [31]. Games by their very nature have the potential to engage the user in game play that can be rewarding through scoring points or following story arcs that can help improve user involvement and lower attrition rates [32,33]. Additionally, as the technology improves, gaming can utilize accessories to provide richer sensory environments and immersive user experiences that allow users to simulate real-life scenarios more safely and help in educating and achieving cognitive and behavioral changes through overlearning and repetition [33,34].

### Research Gap and Aim

Many studies have assessed the effectiveness of serious games to alleviate depression. Aggregating the evidence from these studies is very important to draw more definitive conclusions about the effectiveness of serious games as viable therapeutic interventions in depressive disorders. Several published reviews have summarized the evidence about the effectiveness of serious games for depression [18,19,35-37]. However, these reviews are undermined by certain technical shortcomings that limit the generalization of the findings. Specifically, these reviews (1) focused on a certain type of serious games (ie, exergames) [19,37]; (2) focused on a certain age group (older adults) [37]; (3) included non-randomized controlled trials (RCTs) [19,35]; (4) did not search technical databases (such IEEE Xplore and the ACM Digital Library), thereby including only a few studies [35-37]; (5) did not assess the quality of the evidence [18,19,35-37]; and (6) were outdated publications [18,19,35]. Therefore, this study aimed to assess the effectiveness of serious games for alleviating depression by summarizing and pooling the results of previous studies and providing an up-to-date review.

## Methods

We conducted a systematic review in accordance with the PRISMA (Preferred Reporting Items for Systematic Reviews and Meta-Analyses) statement (Multimedia Appendix 1) [38].

### Search Strategy

#### Search Sources

We utilized the following bibliographic databases to retrieve relevant studies: MEDLINE (via Ovid), PsycInfo (via EBSCO), CINAHL (via EBSCO), IEEE Xplore, ACM Digital Library, and Scopus. These databases were searched on March 30, 2021 by the first author. When applicable, we set auto alerts to conduct an automatic search weekly for 12 weeks (ending on June 30, 2021). We also searched the search engine “Google Scholar” to identify grey literature. We checked only the first 10 pages (ie, 100 hits) because Google Scholar retrieved a vast number of studies and it ordered them based on their relevancy. To identify further studies of relevance to the review, we conducted backward reference list checking (ie, screening the reference lists of the included studies and relevant reviews) and forward reference list checking (ie, screening the studies that cited the included studies).

#### Search Terms

The search query in this review was developed by consulting 2 experts in digital mental health and by checking systematic reviews of relevance to the review. These terms were chosen based on the target intervention (eg, serious games, exergames, and gamification), target outcome (eg, depression and melancholy), and target study design (eg, RCT and clinical trial). Multimedia Appendix 2 shows the detailed search query used to search each of the aforementioned databases.

### Study Eligibility Criteria

This review included only RCTs that assessed the effectiveness of serious games for alleviating the severity of depressive symptoms. To be more precise, the intervention of interest in this review was serious games that were delivered on any digital platform such as computers, consoles (eg, Xbox, PlayStation), mobile phones, tablets, handheld devices, or any other computerized devices. The intervention had to utilize elements of gaming as an integral and primary method for therapeutic or prevention purposes. We did not consider nondigital games and those used for other purposes such as monitoring, screening, and diagnosis. We included RCTs whether they were parallel RCTs, cluster RCTs, crossover RCTs, or factorial RCTs but we excluded quasi-experiments, observational studies, and reviews. We focused on studies in which one of the measured outcomes was depression or depressive symptoms regardless of the outcome measures. Only trials in the English language were eligible for inclusion in this review. RCTs published as journal articles, conference proceedings, and dissertations were included, whereas we excluded conference abstracts and posters, commentaries, preprints, proposals, and editorials. We did not apply restrictions related to the population, year of publication, country of publication, comparator, and study settings.

### Study Selection

We followed 3 steps to identify the relevant studies. In the first step, we exported the retrieved studies to EndNote to identify and remove duplicates. Then, 2 reviewers (EA and MA) independently screened the titles and abstracts of all retrieved studies. In the last step, the 2 reviewers independently screened the full texts of studies included from the second step. A third reviewer (AA) resolved any disagreements between the 2 reviewers in the second and third steps. Cohen κ in this review indicated a very good level of interrater agreement in the first (0.85) and second (0.90) steps [39].

### Data Extraction

Two reviewers (EA and MA) independently extracted data from the included reviews using Microsoft Excel (Microsoft Corporation, Redmond, WA). Multimedia Appendix 3 shows the data extraction form that was used by the 2 reviewers to extract the data precisely and systematically from the included studies. The form was pilot tested using 3 included studies. Any disagreements between the reviewers were resolved by consulting a third reviewer (AA). The interrater agreement between the reviewers was 0.87, indicating a very good level of agreement [39]. Some outcome data (eg, mean, standard deviation, sample size in each group) were missing in 10 studies. Therefore, we contacted their corresponding authors to get them, and 5 corresponding authors did not reply to our emails even after sending 2 reminders.

### Risk of Bias Assessment

Two reviewers (EA and MA) independently assessed the risk of bias in the included studies using the Risk-of-Bias 2 (RoB 2) tool, which is recommended by the Cochrane Collaboration [40]. This tool appraises the risk of bias in 5 domains in RCTs: randomization process, deviations from intended interventions, missing outcome data, measurement of the outcome, and selection of the reported result [40]. The risk of bias judgments in these domains is used to determine the overall risk of bias for each included study. A third reviewer (AA) resolved any disagreements in judgments between the 2 reviewers. Interrater agreement between the reviewers was very good (Cohen κ=0.93) [39].

### Data Synthesis

We utilized narrative and statistical approaches to synthesize the extracted data. Specifically, in narrative synthesis, texts and tables were used to describe the characteristics of the included studies, population, intervention, comparator, and outcome measures. Then, we grouped and summarized the findings of the included studies according to the type of serious games (ie, exergames or computerized CBT games). A meta-analysis was conducted when at least 2 studies of the same type of serious game reported enough data for the analysis (ie, mean, standard deviation, number of participants in each intervention group). When this information was not reported in any included study, we contacted the first and corresponding authors to get the missing information.

Review Manager (RevMan 5.4) was used to conduct the meta-analysis. We measured the effect of each trial and the overall effect using the standardized mean difference (SMD; Cohen *d*) because the outcome data (severity of depressive symptoms) were continuous and tools used to measure the outcome were different between the included studies. The random effects model was used in the analysis given the clinical heterogeneity between the meta-analyzed studies in terms of serious game characteristics (eg, types, duration, frequency, and period), population characteristics (eg, sample size, mean age, and health condition), and outcome measures (ie, tools and follow-up period).

When the meta-analysis showed a statistically significant difference between groups, we examined whether this difference was clinically important. A minimal clinically important difference (MCID) is defined as the smallest change in a measured outcome that a patient would consider as worthy and significant and which mandates a change in a patient’s treatment. The MCID boundaries for an outcome were calculated as ±0.5 times the SMD of the meta-analyzed studies.

We checked the characteristics of participants, interventions, comparator, and outcomes in studies included in the meta-analysis to assess their clinical heterogeneity. We also examined the statistical heterogeneity of the meta-analyzed studies by calculating a Chi-square *P* value and I^2^, which measures the statistical significance of heterogeneity and the degree of heterogeneity, respectively. A Chi-square *P* value ≤.05 indicates heterogeneous meta-analyzed studies [41]. The degree of heterogeneity was considered unimportant, moderate, substantial, or considerable when I^2^ was 0%-40%, 30%-60%, 50%-90%, or 75%-100%, respectively [41].

We assessed the overall quality of evidence from the meta-analyses using the Grading of Recommendations Assessment, Development, and Evaluation (GRADE) approach, which assesses the quality of evidence based on 5 domains: risk of bias, inconsistency (ie, heterogeneity), indirectness, imprecision, and publication bias [42]. Two reviewers (CT and AA) independently assessed the overall quality of meta-analyzed evidence, and any disagreements were resolved through discussion and consensus. Interrater agreement between the reviewers was very good (Cohen κ=0.88) [39].

## Results

### Search Results

**Figure 1:**
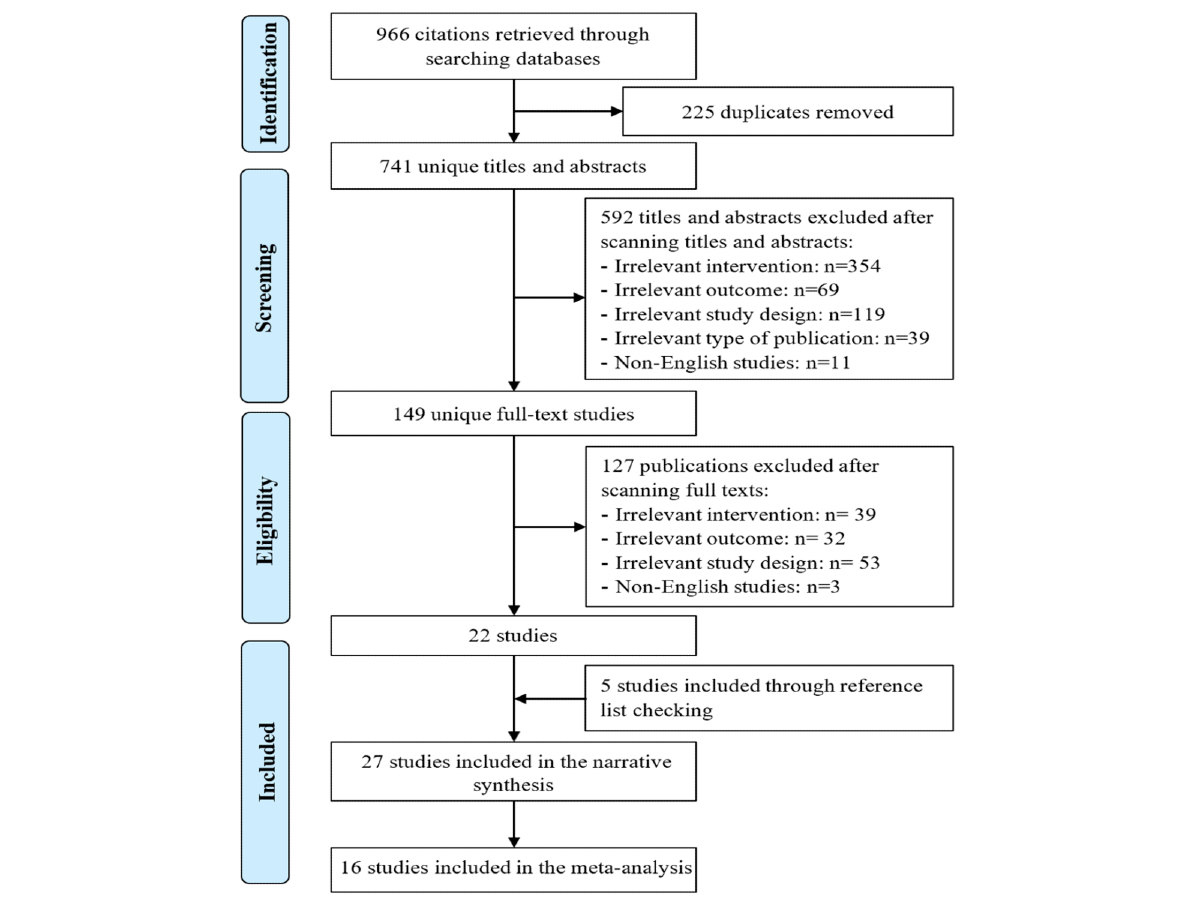
Flow chart of the study selection process.

As shown in Figure 1, we retrieved 966 citations from searching the 7 electronic databases. Using the software EndNote, we identified and removed 225 duplicates of the retrieved citations. Screening titles and abstracts of the remaining 741 citations led to excluding 592 citations because (1) they did not use serious games (n=354); (2) the severity of depressive symptoms was not a measured outcome (n=69); (3) they were not RCTs (n=119); (4) they were not peer-reviewed articles, theses, nor conference proceedings (n=39); and (5) they were published in non-English languages (n=11). Reading the full text of the remaining 149 publications led to excluding 127 publications because (1) they did not use serious games (n=39), (2) the severity of depressive symptoms was not a measured outcome (n=32), (3) they were not RCTs (n=53), and (4) they were published in non-English languages (n=3). We identified 5 additional RCTs through backward and forward reference list checking. In total, 27 RCTs were included in the current review [43-69]. Of those, 16 RCTs were included in the meta-analyses [45-52,54,59-65].

### Characteristics of Included Reviews

The included studies were published between 2012 and 2021 (Table 1). The years that witnessed the largest number of included studies were 2018 (n=5) and 2020 (n=5). The included studies were carried out in 15 different countries, as shown in Table 1. The country that published the largest number of the included studies was Germany (n=5). All included papers were papers published in peer-reviewed journals. The trial type used in the most included studies was parallel RCTs (n=24).

**Table 1:** Characteristics of studies and population.

Author(s), year	Country	Publication type	RCT^a^ type	Sample size, n	Mean age (years)	Sex (male), %	Health condition	Setting
Ruivo et al [43], 2017	Ireland	Journal article	Parallel	32	59.9	81.3	Cardiovascular diseases	Clinical, community, educational
Ferraz et al [44], 2018	Brazil	Journal article	Parallel	62	69	59.7	Parkinson disease	Clinical
Song and Park [45], 2015	South Korea	Journal article	Parallel	40	50.1	55.0	Stroke	Clinical
Schumacher et al [46], 2018	Germany	Journal article	Parallel	42	56.3	59.5	Hematopoietic stem cell transplantation recipients	Clinical
Meldrum et al [47], 2015	Ireland	Journal article	Parallel	71	54.1	38.0	Unilateral peripheral vestibular loss	Clinical
Zhou et al [48], 2020	Qatar	Journal article	Parallel	73	64.6	45.2	Diabetes and end-stage renal disease	Clinical
Vieira et al [49], 2017	Portugal	Journal article	Parallel	46	57.7	-^b^	Coronary artery disease	Clinical
Tollár et al [50], 2018	Hungary	Journal article	Parallel	74	69.4	48.6	Parkinson disease	Clinical
Ozdogar et al [51], 2020	Turkey	Journal article	Parallel	60	40.1	27.1	Multiple sclerosis	Clinical
Kempf and Martin [52], 2013	Germany	Journal article	Parallel	220	61.1	45.9	Type 2 diabetes	Clinical, community
Rendon et al [53], 2012	United States	Journal article	Parallel	40	84.5	35.0	Older adults	Clinical
Jahouh et al [54], 2021	Spain	Journal article	Parallel	80	84.2	44.0	Older adults	Clinical
Rica et al [55], 2019	Brazil	Journal article	Parallel	50	-	0.0	Older adults	Clinical, community
Andrade et al [56], 2020	Brazil	Journal article	Cluster	140	9.41	42.1	Elementary students	Educational
Shin et al [57], 2015	South Korea	Journal article	Parallel	35	54	75	Stroke	Clinical
Adomavičienė et al [58], 2019	Lithuania	Journal article	Parallel	60	64.6	66.7	Stroke	Clinical
Fleming et al [59], 2012	New Zealand	Journal article	Crossover	32	14.9	56	Depression	Educational
Merry et al [60], 2012	New Zealand	Journal article	Parallel	187	15.6	34.2	Depression	Clinical, educational
Donker et al [61], 2019	Netherlands	Journal article	Parallel	193	41.3	33.2	Acrophobia	Community
Perry et al [62], 2017	Australia	Journal article	Cluster	540	16.7	36.9	Secondary students	Educational
Cooney et al [63], 2017	Ireland	Journal article	Parallel	52	40.6	38.8	Anxiety, depression, and/or intellectual disability	Clinical
Poppelaars et al [64], 2016	Netherlands	Journal article	Parallel	208	13.4	0.0	Depression	Educational
Välimäki et al [65], 2018	Finland	Journal article	Parallel	90	41	50.0	Traumatic brain injury	Clinical
Wijnhoven et al [66], 2020	Netherlands	Journal article	Parallel	109	11.1	77.1	Anxiety and autism spectrum disorder	Clinical, educational
Haberkamp et al [67], 2021	Germany	Journal article	Parallel	68	22.8	13.0	Arachnophobia	Educational
Butler et al [68], 2020	Germany	Journal article	Parallel	40	33.4	100	Posttraumatic stress disorder	Clinical
David et al [69], 2018	Germany	Journal article	Parallel	165	12.9	35.9	Stroke	Educational

^a^RCT: randomized controlled trial.

^b^Not reported.

The sample size in the included studies varied from 32 to 540, with an average of 104. The mean age of participants in the included studies ranged between 9.41 years and 84.5 years, with an average of 43.9 years. The percentage of the sample who were men reported in 26 studies ranged from 0% to 100%, with an average of 46.1%. Participants’ health conditions were varied between studies, and depression and stroke were the most common (n=4 each). Participants in most studies were recruited from clinical settings (n=20).

The intervention in the included studies was only serious games in 19 studies, serious games plus occupational therapy in 2 studies, and serious games plus psychotherapy in 1 study (Table 2). The most common games used in the included studies were SPARX (n=4) and Wii Fit (n=4). There were 5 types of serious games based on the therapeutic modality that they deliver: exergames (n=16), computerized CBT games (n=8), exposure therapy games (n=1), brain training games (n=1), rational emotive behavioral therapy (REBT) and rational emotive behavioral therapy education (REBE)–based game (n=1). Although games were designed with a “serious” purpose from the beginning (designed serious games) in 14 studies, they were not designed as serious games but were being used for a serious purpose (purpose-shifted games) in the remaining 13 studies. The most common platforms used for playing the games were computers (n=12) and video game consoles and their accessories (eg, balance board; n=12). The duration of the games in the included studies ranged between 5 minutes and 85 minutes, but it ranged between 20 minutes and 45 minutes in most studies (n=14). The frequency of playing the games varied between once a week and once a day, but it ranged between once a week and 3 times a week in 20 studies. The period of the intervention varied between 1 week and 24 weeks, but it ranged from 4 to 8 weeks in 19 studies.

**Table 2:** Characteristics of interventions.

Author(s)	Intervention	Serious game name	Serious game type	Serious game genre	Platform	Duration (minutes)	Frequency (times/week)	Period (weeks)
Ruivo et al [43]	Serious game	Wii-Sports	Exergame	Purpose-shifted	Wii console and Kinect	60	2	6
Ferraz et al [44]	Serious game	Kinect Adventures	Exergame	Purpose-shifted	Xbox console and Kinect	50	3	8
Song and Park [45]	Serious game	Kinect Sport, Kinect Sport Season 2, Kinect Adventure, and Kinect Gunstringer	Exergame	Purpose-shifted	Computer and Xbox Kinect	30	5	8
Schumacher et al [46]	Serious game	Wii Fit and Wii-Sports	Exergame	Purpose-shifted	Wii console and balance board	30	5	2
Meldrum et al [47]	Serious game	Wii Fit Plus	Exergame	Purpose-shifted	Wii console and balance board and Frii Board	15	5	6
Zhou et al [48]	Serious game	N/R^a^	Exergame	Designed	Computer and wearables (sensors)	30	3	4
Vieira et al [49]	Serious game	Kinect-RehabPlay	Exergame	Designed	Computer and Xbox Kinect	70-85	3	24
Tollár et al [50]	Serious game	Reflex Ridge, Space Pop, Just Dance	Exergame	Purpose-shifted	Xbox console and Kinect	60	5	5
Ozdogar et al [51]	Serious game	Kinect Sports Rivals	Exergame	Purpose-shifted	Xbox console and Kinect	45	1	8
Kempf and Martin [52]	Serious game	Wii Fit Plus	Exergame	Purpose-shifted	Wii console and balance board	≥30	1	12
Rendon et al [53]	Serious game	Wii Fit	Exergame	Purpose-shifted	Wii console and balance board	35-45	3	6
Jahouh et al [54]	Serious game	Step, Nodding	Exergame	Purpose-shifted	Wii console	40-45	2-3	8
Rica et al [55]	Serious game	Kinect Sports Ultimate Collection, Your Shape Fitness Evolved, Dance Central, and Kinect Training	Exergame	Purpose-shifted	Xbox console and Kinect	60	3	12
Andrade et al [56]	Serious game	Just Dance 2015	Exergame	Purpose-shifted	Xbox console and Kinect	40	2	1
Shin et al [57]	Serious game + occupational therapy	RehabMaster	Exergame	Designed	Computer, sensors, and infrared projector	60	5	4
Adomavičienė et al [58]	Serious game	N/R	Exergame	Designed	Computer and Kinect	45	Once a day	2
Fleming et al [59]	Serious game	SPARX	Computerized CBT^b^ game	Designed	Computer	30	1-2	5
Merry et al [60]	Serious game	SPARX	Computerized CBT game	Designed	Computer	20-40	1-2	4-7
Donker et al [61]	Serious game	ZeroPhobia	Computerized CBT game	Designed	Smartphone and wearables (VR^c^ goggles)	5-40	2	3
Perry et al [62]	Serious game	SPARX-R	Computerized CBT game	Designed	Computer	20-30	1-2	5-7
Cooney et al [63]	Serious game	Pesky Gnats: The Feel Good Island	Computerized CBT game	Designed	Computer	60	1	7
Poppelaars et al [64]	Serious game	SPARX	Computerized CBT game	Designed	Computer	20-40	1	7
Välimäki et al [65]	Serious game	CogniFit	Computerized CBT game	Designed	Computer	≥30	Once a day	8
Wijnhoven et al [66]	Serious game	MindLight	Computerized CBT game	Designed	Computer and wearable (headset)	60	1	6
Haberkamp et al [67]	Serious game	Spider App	Exposure therapy game	Designed	Smartphone	12	2	1
Butler et al [68]	Serious game + psychotherapy	Tetris	Brain-training game	Purpose-shifted	Nintendo DS XL console	60	2	6
David et al [69]	Serious game	REThink	REBT^d^- and REBE^e^-based game	Designed	Tablet	50	3	4

^a^N/R: not reported.

^b^CBT: cognitive behavioral theory.

^c^VR: virtual reality.

^d^REBT: rational emotive behavioral therapy.

^e^REBE: rational emotive behavioral education.

As shown in Table 3, the comparison groups received inactive interventions in 15 studies, while they received active interventions in 18 studies (eg, conventional exercises, CBT programs, video games, and psychotherapy). Note that the numbers do not add up because 6 studies delivered both active and inactive interventions as comparators. The duration of the active comparators ranged between 12 minutes and 100 minutes. The frequency of playing the active comparators varied between once a week and once a day. The period of the active comparators varied between 1 week and 24 weeks. The outcome of interest (eg, severity of depressive symptoms) was measured using 18 different tools, but the most common tool used by the included studies was the Beck Depression Inventory (BDI; n=6), followed by the Hospital Anxiety and Depression Scale (HADS; n=4). The outcome of interest was measured immediately after the intervention in all included studies, and the most common follow-up period was 3 months (n=6). Participant attrition was reported in 24 studies and ranged from 0 to 134.

**Table 3:** Characteristics of comparators and outcomes.

Author(s)	Comparator	Duration (minutes)	Frequency (times/week)	Period (weeks)	Outcome measures	Follow up	Attrition, n
Ruivo et al [43]	Conventional exercises (functional training), conventional exercises (bicycle exercise)	50	3	8	GDS^a^	Postintervention	10
Ferraz et al [44]	Robot-assisted trainings	45	Once a day	2	HADS^b^	Postintervention	18
Song and Park [45]	Conventional exercises	30	5	8	BDI^c^	Postintervention	N/R^d^
Schumacher et al [46]	Conventional exercises	30	5	2	HADS-D^e^	Postintervention, 30-day follow-up, 100-day follow-up	11
Meldrum et al [47]	Conventional exercises	15	5	6	HADS-D	Postintervention	9
Zhou et al [48]	Conventional exercises	30	3	4	CES-D^f^	Postintervention	0
Vieira et al [49]	Conventional exercises, control	70-85	3	24	DASS-21^g^	Postintervention, mid-intervention (3 months)	13
Tollár et al [50]	Conventional exercises	60	2	6	HADS	Postintervention, 2-month follow-up	4
Ozdogar et al [51]	Conventional exercises, control	45	1	8	BDI	Postintervention	3
Kempf and Martin [52]	Control	N/A^h^	N/A	N/A	WHO-5^i^, PAID^j^, ADS-L^k^	Postintervention	44
Rendon et al [53]	Control	N/A	N/A	N/A	GDS	Postintervention	6
Jahouh et al [54]	Control	N/A	N/A	N/A	GDS, GADS^l^	Postintervention	N/R
Rica et al [55]	Conventional exercises, control	60	5	5	BDI	Postintervention	0
Andrade et al [56]	Physical education	40	2	1	BMS^m^	Postintervention	0
Shin et al [57]	Occupational therapy	60	5	4	HAMD^n^	Postintervention	3
Adomavičienė et al [58]	Conventional exercises, control	60	5	5	BDI	Postintervention	0
Fleming et al [59]	Control	N/A	N/A	N/A	CDRS-R^o^, RADS-2^p^	Postintervention	5
Merry et al [60]	Control	N/A	N/A	N/A	CDRS-R, RADS-2	Postintervention, 3-month follow-up	17
Donker et al [61]	Control	N/A	N/A	N/A	PHQ-9^q^	Postintervention, 3-month follow-up	59
Perry et al [62]	Interactive online program	20-30	1-2	5-7	MDI^r^	Postintervention, 6-month follow-up, 18-month follow-up	134
Cooney et al [63]	Control	N/A	N/A	N/A	GAS-LD^s^	Postintervention, 3-month follow-up	3
Poppelaars et al [64]	CBT^t^ program + serious game, CBT program, control	CBT program + serious game (80-100), CBT program (60)	1	7	RADS-2	Postintervention, 3-month follow-up, 6-month follow-up, 12-month follow-up	10
Välimäki et al [65]	Video game, control	≥30	Once a day	8	PHQ-9	Postintervention, 3-month follow-up	20
Wijnhoven et al [66]	Video game	60	1	6	CDI-2^u^	Postintervention, 3-month follow-up	35
Haberkamp et al [67]	Video game	12	2	1	BDI-II	Postintervention, 2-week follow-up	6
Butler et al [68]	Psychotherapy	60	2	6	BDI-II	Postintervention, 6-month follow-up	0
David et al [69]	Rational emotive behavioral therapy and education, control	50	3	4	EATQ-R^v^	Postintervention	23

^a^GDS: Geriatric Depression Scale.

^b^HADS: Hospital Anxiety and Depression Scale.

^c^BDI: Beck Depression Inventory.

^d^N/R: not reported.

^e^HADS-D: depression subscale of the HADS.

^f^CES-D: Center for Epidemiologic Studies Depression Scale.

^g^DASS-21: Depression, Anxiety and Stress Scale 21.

^h^N/A: not applicable.

^i^WHO-5: WHO 5-item Well-Being Index.

^j^PAID: Problem Areas in Diabetes.

^k^ADS-L: Allgemeine Depressionsskala.

^l^GADS: Goldberg Anxiety and Depression Scale.

^m^BMS: Brunel’s Mood Scale.

^n^HAMD: Hamilton Depression Rating Scale.

^o^CDRS-R: Children’s Depression Rating Scale-Revised.

^p^RADS-2: Reynolds Adolescent Depression Scale.

^q^PHQ-9: Patient Health Questionnaire-9.

^r^MDI: Major Depression Inventory.

^s^GAS-LD: Glasgow Depression Scale for people with a learning disability.

^t^CBT: cognitive behavioral therapy.

^u^CDI-2: Child Depression Inventory 2.

^v^EATQ-R: Early Adolescent Temperament Questionnaire-Revised.

### Results of Risk of Bias Appraisal

**Figure 2:**
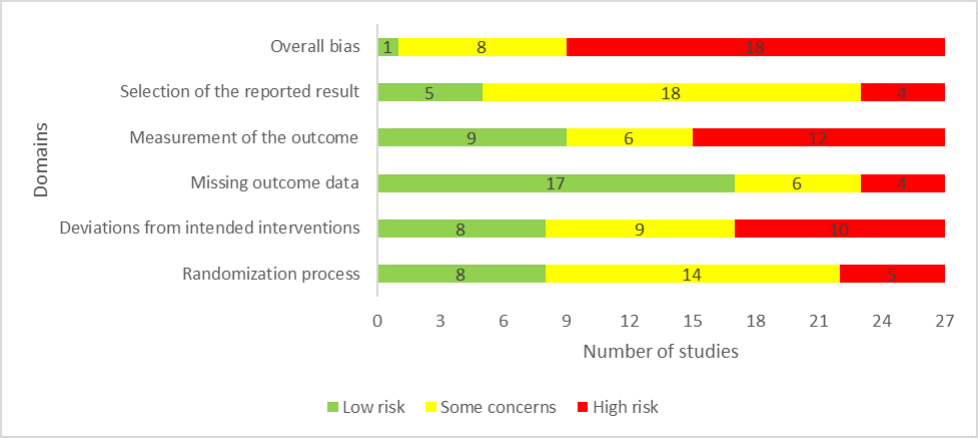
Review authors’ judgments about each “risk of bias” domain.

The random allocation sequence for the randomization process was appropriate in 23 included studies. However, only 10 studies concealed the allocation sequence until participants were enrolled and assigned to interventions, and groups were not comparable in 4 studies. Accordingly, the risk of bias due to the randomization process was rated as low for only 8 studies (Figure 2).

Participants and individuals delivering the interventions were aware of assigned interventions during the experiment in 22 and 20 studies, respectively. Deviation from the intended intervention occurred in 2 studies due to the experimental contexts. Only 14 studies used an appropriate analysis (intention-to-treat or modified intention-to-treat analyses) to estimate the effect of assignment to intervention. Therefore, the risk of bias due to the deviations from the intended interventions was judged as low in only 8 studies (Figure 2).

Outcome data were not available for all or nearly all participants in 21 studies, and there was evidence that the findings were not biased by missing outcome data in only 5 studies. The reasons for missing outcome data could not be related to the true value of the outcome in 18 studies. As a result, 17 studies were judged as having a low risk of bias in the “missing outcome data” domain.

All included studies assessed the outcome of interest (ie, depression level) using appropriate measures and used measurement methods comparable across intervention groups. However, the assessor of the outcome was blinded in only 9 studies. For this reason, only these studies were rated as low risk of bias in the “measuring the outcome” domain (Figure 2).

In 17 studies, a prespecified analysis plan (ie, protocol) was not published. Only 4 studies reported outcome measurements different from those specified in the analysis plan. There is no evidence that all included studies selected their results from many results produced from multiple eligible analyses of the data. Accordingly, the risk of bias due to the selection of the reported results was considered low in 4 studies (Figure 2).

In the last domain “overall bias,” the risk of bias was considered high in 20 studies as they were judged as having a high risk of bias in at least one domain; 6 studies were judged to have some concerns in the domain of overall bias as they had some concerns in at least one of the domains and were not at high risk for any domain. The remaining study was judged to be at low risk of bias for the domain of overall bias given that it was rated to be at low risk of bias for all domains. Reviewers’ judgments about each “risk of bias” domain for each included study are presented in Multimedia Appendix 4.

### Results of Studies

#### Types of Serious Games

As mentioned earlier, we identified 5 types of serious games based on the therapeutic modality that they deliver in the included studies. The first type is exergames, which refer to video games that require physical exercises (eg, fitness and balance exercises) in order to be played. The second type is computerized CBT games, which are video games that provide CBT for the users. The third type is exposure therapy games, which are video games that apply exposure principles to reduce anxiety in users with phobias. The fourth type is brain training games, which are video games that are based on cognitive interference tasks to reconsolidate traumatic memories. The last type is REBT- and REBE-based games, which are video games that enable users to replace irrational beliefs (eg, demandingness, intolerance, and frustration) with rational beliefs (eg, unconditional acceptance and tolerance). Results of the included studies were grouped into 3 categories based on the types of serious games.

#### Exergames

Exergames were the intervention in 16 studies [43-58]. Exergames were compared with conventional exercises, no intervention, physical education, and occupational therapy. The results of these comparisons are summarized in the following sections.

##### Exergames Versus Conventional Exercises

In 9 studies, the effect of exergames was compared with that of conventional exercises on the severity of depressive symptoms [43-51]. Although 1 study did find a statistically significant difference in the severity of depressive symptoms between the groups [45], the remaining 8 studies did not [43,44,46-51]. Specifically, Song and Park [45] compared the effect of exergames (Kinect Sport, Kinect Sport Season 2, Kinect Adventure, and Kinect Gunstringer) with that of conventional exercises (ergometer bicycle training) on the severity of depressive symptoms (measured using the BDI) among patients with stroke. The study found a statistically significant difference (*P*<.05) in depressive symptoms between the groups, favoring exergames over ergometer training. Another study assessed the effect of exergames (Wii Fit Plus) on the severity of depressive symptoms (measured using the HADS-D) among patients with unilateral peripheral vestibular loss [47]. The study did not find any statistically significant difference (*P*=.49) in the severity of depressive symptoms between the exergame group and conventional exercise group [47]. Schumacher et al [46] assessed the effects of exergames (Wii Fit and Wii-Sports) and conventional exercises on depression symptoms (measured using the HADS-D) among hematopoietic stem cell transplantation recipients and found no significant difference (*P*=.07) between both groups in the severity of depressive symptoms. A study by Ozdogar et al [51] examined the effects of exergames (Kinect Sports Rivals) and conventional exercises on the severity of depressive symptoms among patients with multiple sclerosis, and there was no significant difference (*P*>.05) in the severity of depressive symptoms between the 2 groups. A study examining the effect of exergames (Kinect-RehabPlay) on the severity of depressive symptoms (measured using the BDI) among patients with coronary artery disease in comparison with conventional exercises found no significant difference in the severity of depressive symptoms between the 2 groups [49]. In another study [48], no significant difference in the severity of depressive symptoms (measured using the Center for Epidemiologic Studies Depression Scale in patients with diabetes and end-stage renal disease) was detected between the exergame group and conventional exercise group. Tollár et al [50] compared the effect of exergames (Reflex Ridge, Space Pop, Just Dance) with that of conventional exercises (ergometer bicycle training) on the severity of depressive symptoms (measured using the BDI) among patients with Parkinson disease. The study showed no statistically significant difference (*P*=.27) in the severity of depressive symptoms between the 2 groups. A study assessed the effects of exergames (Wii-Sports) and conventional exercises on the severity of depressive symptoms (measured by HADS) among patients with a high risk of cardiovascular diseases [43]. No statistically significant difference between the groups was reported in the study [43]. In the last study, no significant difference in the severity of depressive symptoms (measured using the Geriatric Depression Scale [GDS] in patients with Parkinson disease) was detected between the exergame group (Kinect Adventures) and 2 conventional exercise groups (functional training and bicycle exercises) [44].

**Figure 3:**
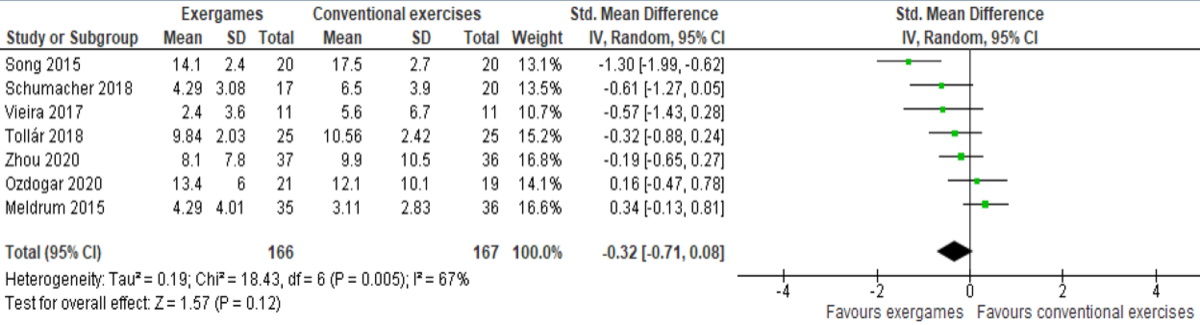
Forest plot of 7 studies comparing the effect of exergames with that of conventional exercises on the severity of depressive symptoms.

Results of 7 studies were meta-analyzed as shown in Figure 3 [45-49,51]. No statistically significant difference (*P*=.12) in the severity of depressive symptoms was found between the exergame group and conventional exercise group (SMD –0.32, 95% CI –0.71 to 0.08). There was substantial heterogeneity in the evidence (*P*=.005; I^2^=67%). The quality of the evidence was very low, as it was downgraded by 5 levels due to a high risk of bias, heterogeneity, and imprecision (Multimedia Appendix 5).

##### Exergames Versus No Intervention

In 7 studies, the effect of exergames was compared with that of no intervention on the severity of depressive symptoms [49-55]. Although 4 studies showed a statistically significant difference in the severity of depressive symptoms between the groups [50-52,55], 3 studies did not [49,53,54]. Specifically, Kempf and Martin [52] compared the effect of exergames (Wii Fit Plus) with that of no intervention on the severity of depressive symptoms (measured using the WHO 5-item Well-Being Index [WHO-5], Problem Areas in Diabetes [PAID], and Allgemeine Depressionsskala [ADS-L]) in patients with type 2 diabetes. The study found a statistically significant effect of exergames over no intervention on the severity of depressive symptoms as measured using the WHO-5 (*P*<.001), PAID (*P*=.007), and ADS-L (*P*=.002) [52]. A study conducted by Ozdogar et al [51] examined the effects of exergames (Kinect Sports Rivals) and no intervention on the severity of depressive symptoms (measured using the BDI) among patients with multiple sclerosis. Interestingly, the study demonstrated a statistically significant difference (*P*<.05) between the groups, favoring no intervention over exergames [51]. In another study [55], the influence of exergames (Kinect Sports Ultimate Collection, Your Shape Fitness Evolved, Dance Central, Kinect Training) and no intervention on the severity of depressive symptoms (measured using the BDI) among older women was investigated, and a statistically significant difference in the severity of depressive symptoms between groups was detected, favoring exergames over no intervention. Tollár et al [50] compared the effect of exergames (Reflex Ridge, Space Pop, Just Dance) with that of no intervention on the severity of depressive symptoms (measured using the BDI) among patients with Parkinson disease. The study showed a statistically significant difference (*P*<.001) in the severity of depressive symptoms between the 2 groups, favoring exergames over no intervention. Jahouh et al [54] assessed the effect of exergames (Wii Fit game) on the severity of depressive symptoms (measured using the GDS and Goldberg Anxiety and Depression Scale [GADS]) among older adults. No significant difference in the severity of depressive symptoms as measured using the GDS (*P=*.43) and GADS (*P=*.21) was detected between the exergame group and the control group [54]. Another study examining the effect of exergames (Kinect-RehabPlay) on the severity of depressive symptoms (measured using the BDI) among patients with coronary artery disease in comparison with no intervention found no significant difference (*P*>.05) in the severity of depressive symptoms between the 2 groups [49]. The effects of exergames and no intervention on the severity of depressive symptoms (measured by GDS) among older adults were compared in another study [53], and no significant difference (*P*=.09) was found in the severity of depressive symptoms between the 2 groups [53].

**Figure 4:**
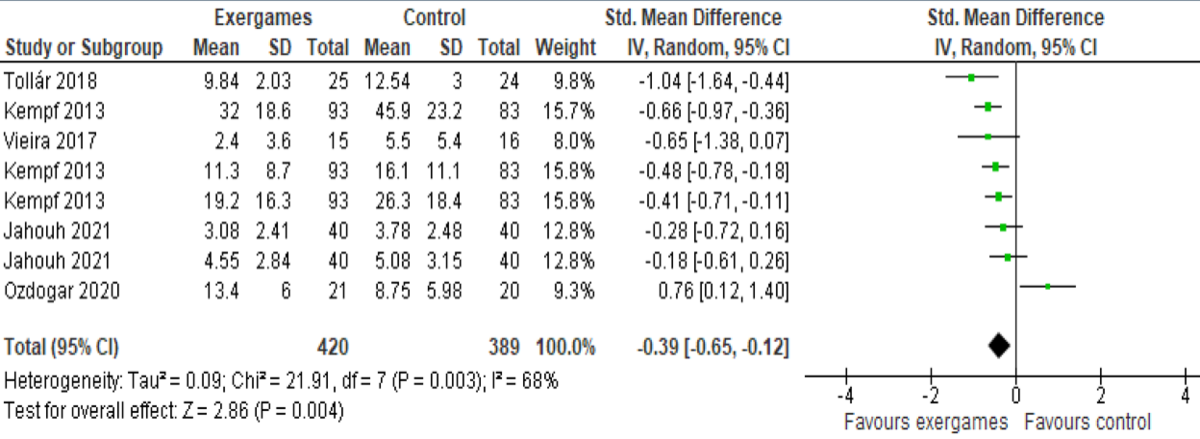
Forest plot of 5 studies (8 comparisons) comparing the effect of exergames with that of no intervention on the severity of depressive symptoms.

We meta-analyzed results of 5 studies, as they reported enough and appropriate data for the analysis [49-52,54]. Of the 5 studies, 2 assessed the severity of depressive symptoms using more than one measure (ie, WHO-5, PAID, and ADS-L [52]; GDS and GADS [54]); therefore, we included the results of all these measures in the meta-analysis. The meta-analysis showed a statistically significant difference in the severity of depressive symptoms (*P*=.004) between exergame and control groups, favoring exergames over no intervention (SMD –0.39, 95% CI –0.65 to –0.12; Figure 4). This difference was also clinically important as the overall effect was outside the MCID boundaries (–0.195 to 0.195) and its CI did not cross the “no effect” line (zero effect) and both MCID boundaries. The statistical heterogeneity of the evidence was substantial (*P*=.003; I^2^=68%). The quality of the evidence was very low, as it was downgraded by 6 levels due to a high risk of bias, heterogeneity, and imprecision (Multimedia Appendix 5).

##### Exergames Versus Other Active Interventions

In 3 studies, the effect of exergames was compared with that of active interventions on the severity of depressive symptoms, and no statistically significant difference was found between the groups [56-58]. To be more precise, the first study examined the effects of an exergame (Just Dance 2015) and physical education on the severity of depressive symptoms (measured with the Brunel Mood Scale) among elementary students and demonstrated no statistically significant difference (*P*=.13) in the severity of depressive symptoms between the exergame group and physical education group [56]. The second study compared the effect of exergames (RehabMaster) with that of occupational therapy on the severity of depressive symptoms (measured using the Hamilton Depression Rating Scale) among patients with stroke. The study found no statistically significant difference (*P*=.56) in the severity of depressive symptoms between the exergame group and occupational therapy group [57]. The third study compared the effect of exergames with that of robot-assisted training on the severity of depressive symptoms (measured using the HADS) among patients with stroke and did not find any statistically significant difference between the groups [58].

#### Computerized CBT Games

Computerized CBT games were the intervention in 8 studies [59-66]. Computerized CBT games were compared with no intervention, video games, and conventional CBT. The results of these comparisons are summarized in the following sections.

##### Computerized CBT Games Versus No Intervention

In 7 studies, the effect of computerized CBT games was compared with that of no intervention on the severity of depressive symptoms [59-65], and 4 of these studies assessed the effect of a computerized CBT game (SPARX) on the severity of depressive symptoms among patients with depression [59,60,64]. The first study found a statistically significant effect of the computerized CBT game over no intervention on the severity of depressive symptoms as measured using the Children’s Depression Rating Scale-Revised (CDRS-R; *P*=.001) but not the Reynolds Adolescent Depression Scale (RADS-2; *P*=.08) [59]. In the second study [62], the effect of a computerized CBT game (SPARX-R) on the severity of depressive symptoms (measured using the Major Depression Inventory) among secondary students was compared with placebo, an interactive online program (LIFESTYLE) that provides information about several topics unrelated to mental health. The study found a statistically significant difference in the severity of depressive symptoms (as measured postintervention, *P*<.001, and at a 6-month follow-up, *P*=0.01) between the groups, favoring SPARX-R over LIFESTYLE [62]. In contrast, the third study found no statistically significant difference between the 2 groups in the severity of depressive symptoms as measured with the CDRS-R (*P*=.26) and RADS-2 (*P*=.16) [60]. Similarly, the fourth study did not show any statistically significant difference (*P*=.96) in the severity of depressive symptoms between the SPARX group and the control group [64]. Another study examined the effects of a computerized CBT game (Pesky Gnats: The Feel Good Island) and no intervention on the severity of depressive symptoms (measured using the Glasgow Depression Scale for people with a learning disability) among patients with anxiety, depression, or intellectual disability [63]. No statistically significant difference (*P*=.25) in the severity of depressive symptoms was detected between the groups [63]. Välimäki et al [65] compared the effect of a computerized CBT game (CogniFit) with that of no intervention on the severity of depressive symptoms (measured using the Patient Health Questionnaire-9 [PHQ-9]) among patients with traumatic brain injury and found no statistically significant difference (*P*=.76) between the groups. In the last study in this comparison, the effects of a computerized CBT game (ZeroPhobia) and no intervention on the severity of depressive symptoms (measured using the PHQ-9) among patients with acrophobia were investigated [61]. No statistically significant difference (*P*=.12) in the severity of depressive symptoms was found between the 2 groups [61].

**Figure 5:**
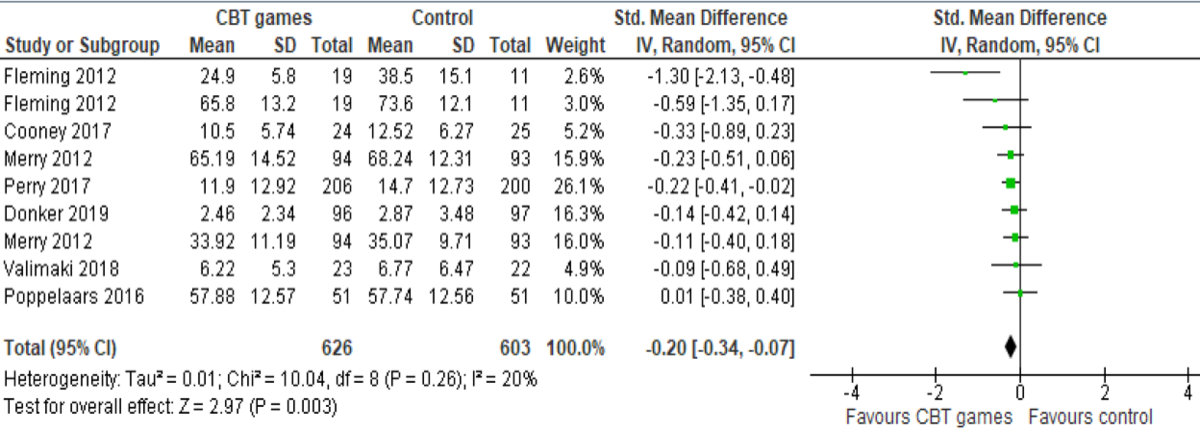
Forest plot of 7 studies (9 comparisons) comparing the effect of CBT games with that of no intervention on depression.

Results of these 7 studies were meta-analyzed, as shown in Figure 5. Because 2 of these studies assessed the severity of depressive symptoms using 2 different measures (CDRS-R and RADS-2), we included the results of both measures of each study in the meta-analysis. The overall effect was statistically significant (*P*=.003) indicating that computerized CBT games are more effective than no intervention in alleviating depressive symptoms: (SMD –0.20, 95% CI –0.34 to –0.07). This difference was also clinically important as the overall effect was outside the MCID boundaries (–0.10 to 0.10) and its CI did not cross the “no effect” line (zero effect) and both MCID boundaries. For this outcome, MCID boundaries were calculated as ±0.5 times the SMD value (–.20). The heterogeneity of the evidence was not a concern (*P*=.26; I^2^= 20%). The quality of the evidence was very low, as it was downgraded by 3 levels due to the high risk of bias and impression (Multimedia Appendix 5).

##### Computerized CBT Games Versus Active Interventions

Välimäki et al [65] compared the effect of a computerized CBT game (CogniFit) with that of entertainment video games on the severity of depressive symptoms (measured using the PHQ-9) among patients with traumatic brain injury and found no statistically significant difference (*P*=.36) between the groups. Another study compared the effect of a computerized CBT game (MindLight) with that of an entertainment video game (Triple Town) on the severity of depressive symptoms (Child Depression Inventory 2) among patients with autism spectrum disorder and anxiety [66]. No statistically significant difference (*P*>.05) in the severity of depressive symptoms was detected between the groups [66]. A study carried out by Poppelaars et al [64] assessed the effects of a computerized CBT game (SPARX) and a conventional CBT program on the severity of depressive symptoms (measured using the RADS-2) among patients with depression. The study did not detect a statistically significant difference (*P*=.58) in the severity of depressive symptoms between the groups.

#### Other Types of Serious Games

One study compared the effect of an exposure therapy game (Spider App) with that of an entertainment video game (Bubble Shooter) on the severity of depressive symptoms (measured using the BDI-II) among patients with arachnophobia [67]. No statistically significant difference (*P*=.95) in the severity of depressive symptoms was detected between the groups [67]. Butler et al [68] examined the effects of brain training games and psychotherapy on the severity of depressive symptoms (measured using the BDI) among patients with posttraumatic stress disorder. No statistically significant difference (*P*=.95) in the severity of depressive symptoms between the 2 groups was detected [68]. In another study, the effect of REBT- and REBE-based games on the severity of depressive symptoms (measured using the Early Adolescent Temperament Questionnaire-Revised) among patients with stroke was compared with conventional REBE and no intervention [69]. The study found a statistically significant difference in the severity of depressive symptoms between the groups, favoring REBT- and REBE-based games over conventional REBE (*P*=.03) and no intervention (*P*<.001).

## Discussion

### Principal Findings

This review assessed the effectiveness of serious games on the severity of depressive symptoms as reported by RCTs. Although 27 RCTs were included in the current review, 16 studies were included in the meta-analysis. Very low-quality evidence from 7 RCTs showed no statistically significant effect of exergames on the severity of depressive symptoms as compared with conventional exercises. Furthermore, 3 studies that compared the effect of exergames with that of other active interventions (eg, occupational therapy and robot-assisted training) on the severity of depressive symptoms and were not included in the meta-analyses found no statistically significant difference between the groups. These findings indicate that exergames are as effective as active interventions, which are usually delivered and supervised by health care providers (eg, physiotherapists, occupational therapists, and psychologists).

Very low-quality evidence from 5 RCTs showed a statistically and clinically significant effect of exergames on the severity of depressive symptoms when compared with no intervention.

Findings in this review are comparable to other reviews. Specifically, a recently published meta-analysis of 5 RCTs conducted by Yen and Chiu [37] showed an overall statistically significant effect (*P*<.001) of exergames on depression. Additionally, another recent meta-analysis of 8 RCTs conducted by Li et al [19] showed a significant effect of exergames on depression. However, both reviews [19,37] compared the effect of exergames with the effects of different active and inactive interventions through one meta-analysis, while our review conducted 2 separate meta-analyses to compare exergames with conventional exercises and no intervention respecting the uniqueness of these 2 interventions. Further, in contrast to our review, 5 of the 8 studies included in the review by Li et al were not RCTs (quasi-experimental or pre-post one-group trials) [19].

Very low-quality evidence from 6 RCTs showed a statistically and clinically significant effect of computerized CBT games on the severity of depressive symptoms when compared with no intervention. In contrast, 3 studies that compared the effect of computerized CBT games with those of active interventions (eg, video games and conventional CBT) on depressive symptoms and were not included in the meta-analyses found no statistically significant difference between the groups. This insignificant effect can be attributed to the fact that conventional CBT is comparable to the active interventions, thereby comparing the effect of 2 comparable interventions usually produces no significant difference, which indicates that computerized CBT games are at least as effective as these active interventions. None of the previous reviews [18,19,35-37] assessed the effect of computerized CBT games on depression.

### Strengths and Limitations

#### Strengths

This review bridged the gaps of previous reviews by focusing on all types of serious games, including only RCTs, targeting all age groups, searching technical databases, assessing the quality of evidence, and synthesizing the data statistically. Therefore, it is more comprehensive than previous reviews [18,19,35-37]. As we followed highly recommended guidelines (ie, PRISMA) to conduct this review, it can be considered a robust and high-quality review.

The risk of publication bias in this review is minimal, as we searched the most popular databases in information technology and health fields; conducted backward and forward reference list checking; used a comprehensive search query; searched grey literature databases; and did not restrict our search to a certain country, year, setting, population, and comparator.

The risk of selection bias in this review is minimal because 2 reviewers independently performed the study selection, data extraction, risk of bias assessment, and quality of evidence evaluation with a very good interrater agreement for all processes. The quality of the evidence was appraised to enable the reader to draw more accurate conclusions. When possible, we synthesized data statistically, and this improved the power of studies and increased the estimates of the likely size of the effect of serious games on depression.

#### Limitations

The intervention of interest in this review was restricted to serious games delivered on any digital platform and used as a therapeutic intervention. Thus, this review cannot comment on the effectiveness of nondigital serious games and those used for other purposes such as monitoring, screening, or diagnosis. The outcome of interest in this review was depression; therefore, we cannot comment on the effectiveness of serious games on other mental health outcomes.

The review was restricted to RCTs written in the English language; therefore, many studies were excluded because they were quasi-experiments or written in other languages. This restriction was necessary because RCTs have higher internal validity than any other study design [70] and owing to practical constraints, it was not feasible to translate all non-English studies.

Most included studies recruited patients without depression; thereby, the effect of serious games on the severity of depression symptoms was not significant. Further, the overall risk of bias was high in most included studies, and the quality of evidence for the meta-analyses was very low. Accordingly, findings in this review must be interpreted with caution.

### Research and Practical Implications

#### Research Implications

Although the severity of depression was one of the measured outcomes in all included studies, only 5 studies recruited patients with depression. This might lead to underestimating the effect of serious games. Therefore, future studies need to recruit participants with depression to assess the effectiveness of serious games on depression.

The therapeutic modalities provided by serious games in most included studies were either exercises or CBT. Further, serious games were not designed specifically to alleviate depression in about half of the studies. Thus, there is a pressing need to assess the effectiveness of serious games that are designed specifically to alleviate depression and deliver other therapeutic modalities such as art therapy, psychotherapy, relaxation-based exercises, psychoeducation, rational emotive behavioral therapy, and exposure therapy, and the list goes on.

Most included studies were carried out in high-income countries; thereby, our findings may not be generalizable to low-income countries. Researchers should conduct more studies to assess the effectiveness of serious games in low-income countries. We excluded many studies that assessed the effectiveness of serious games on other mental disorders such as anxiety and dementia. Further systematic reviews need to be carried out to investigate the effectiveness of serious in alleviating other mental disorders.

The overall risk of bias was high in most included studies mainly due to issues in the randomization process, deviations from the intended outcomes, and selection of the reported result. Further, several studies were not included in the meta-analysis due to missing outcome data. For this reason, we encourage researchers to follow recommended guidelines or tools (eg, RoB 2 [40]) when conducting and reporting RCTs to avoid such biases.

This review hopefully augurs the possible potential of serious games in mental health disorders, but it also underlines that this field, albeit full of potential, is still in its infancy. More studies are needed to prove the significant role of serious games in alleviating depression.

#### Practical Implications

Overall, this study showed that serious games can be effective in alleviating depression in comparison with no intervention, and they can be comparable to other traditional therapeutic interventions for alleviating depressive symptoms. However, findings in this review must be interpreted with caution because the overall risk of bias was high in most included studies, the quality of evidence in the meta-analyses was very low, few studies recruited patients with depression, and serious games in half of the studies were purpose-shifted. Therefore, we can only recommend health care providers consider offering serious games as an adjunct to existing interventions until further, more robust evidence is available.

As mentioned before, serious games in more than half of the studies were not designed to specifically alleviate depression and did not deliver other therapeutic modalities such as art therapy, REBT, and psychoeducation. This may be attributed to the lack of such serious games in real life. Accordingly, there is a need to develop more serious games that are designed to specifically alleviate depression and deliver other therapeutic modalities.

The most common platforms used for playing the games were computers and video game consoles and their accessories, which are relatively more expensive and less accessible than smartphones that were the platform for serious games in only 1 study. The number of smartphone users in the world exceeded 6.4 billion in 2021 [71], which forms about 82% of the global population (7.8 billion) [72]. We encourage developers to develop serious games that can be played through smartphones.

Most studies were carried out in high-income countries, and this may indicate the lack of serious games in low-income countries. People in low-income countries may be more in need of serious games than those in high-income countries because low-income countries have a greater shortage of mental health professionals than high-income countries (0.1 per 1,000,000 people vs 90 per 1,000,000 people) [73,74]. Serious games should be exploited to alleviate depression in low-income countries.

Gaming and mental health have traditionally been two distinctly separate fields and come with their own unique pedagogy and praxis. The potential of utilizing the advantages inherent to gaming, as described earlier, from its reach to its transformative potential in mental health holds a lot of promise in theory. However, to achieve this potential, experts from the two disciplines need to work together in order to understand the unique strengths and limitations of each field when designing serious games.

### Conclusion

Overall, serious games can be better than no intervention in alleviating depression and as effective in alleviating depression as other active interventions (eg, conventional CBT, exposure therapy, conventional exercise). However, definitive conclusions regarding the effectiveness of serious games could not be drawn in this review because the overall risk of bias was high in most included studies, the quality of the meta-analyzed evidence was very low, and few studies recruited patients with depression. Therefore, we can only recommend health care providers consider offering serious games as an adjunct to existing interventions until further, more robust evidence is available. To have sufficient evidence, future studies should assess the effectiveness of serious games that are designed specifically to alleviate depression and deliver other therapeutic modalities, recruit participants with depression, and avoid biases by following recommended guidelines for conducting and reporting RCTs (eg, RoB 2).

## Appendix

Multimedia Appendix 1 Preferred Reporting Items for Systematic Reviews and Meta-Analyses (PRISMA) checklist.

Multimedia Appendix 2 Search strategy.

Multimedia Appendix 3 Data extraction form.

Multimedia Appendix 4 Reviewers’ judgements about each “risk of bias” domain for each included study.

Multimedia Appendix 5 Grading of Recommendations Assessment, Development and Evaluation (GRADE) Profile for comparison of serious games to control or conventional exercises for depression.

## Abbreviations

ADS-L: Allgemeine Depressionsskala
BDI: Beck Depression Inventory
CBT: Cognitive behavioral therapy
CDRS-R: Children’s Depression Rating Scale-Revised
GADS: Goldberg Anxiety and Depression Scale
GDS: Geriatric Depression Scale
GRADE: Grading of Recommendations Assessment, Development and Evaluation
HADS: Hospital Anxiety and Depression Scale
MCID: minimal clinically important difference
PAID: Problem Areas in Diabetes
PHQ-9: Patient Health Questionnaire-9
PRISMA: Preferred Reporting Items for Systematic Reviews and Meta-Analyses
RADS-2: Reynolds Adolescent Depression Scale
RCT: randomized controlled trial
REBE: rational emotive behavioral education
REBT: rational emotive behavioral therapy
RoB 2: Risk-of-Bias 2
SMD: standardized mean difference
WHO: World Health Organization
WHO-5: WHO 5-item Well-Being Index
