# Supplementary material for: The Effectiveness of Serious Games for Alleviating Depression: Systematic Review and Meta-analysis
Source: JMIR Serious Games. 2022 Jan 14;10(1):e32331. doi: 10.2196/32331 (PMC8800090; doi:10.2196/32331)
Supplement: Multimedia Appendix 2 [file games_v10i1e32331_app2.docx]

**Appendix 2: search strategy**

Database(s): **Ovid MEDLINE(R) ALL**1946 to March 30, 2021
Search Strategy:

| **#** | **Searches** | **Results** |
| --- | --- | --- |
| 1 | exp Depressive Disorder/ | 111856 |
| 2 | "depressive disorder".tw. | 30542 |
| 3 | exp Depression/ | 125061 |
| 4 | depression.tw. | 347642 |
| 5 | depressed.tw. | 96012 |
| 6 | Melanchol*.tw. | 1422 |
| 7 | 1 or 2 or 3 or 4 or 5 or 6 | 451986 |
| 8 | exp Video Games/ | 5751 |
| 9 | "serious gam*".tw. | 762 |
| 10 | "game-based".tw. | 659 |
| 11 | "videogam*".tw. | 767 |
| 12 | "video game*".tw. | 3469 |
| 13 | gamification.tw. | 583 |
| 14 | gamified.tw. | 304 |
| 15 | exergam*.tw. | 691 |
| 16 | "Applied game*".tw. | 19 |
| 17 | virtual reality game*.tw. | 114 |
| 18 | Virtual reality-based game*.tw. | 3 |
| 19 | augmented reality game*.tw. | 29 |
| 20 | augmented reality-based game*.tw. | 0 |
| 21 | 7 or 8 or 9 or 10 or 11 or 12 or 13 or 14 or 15 or 16 or 17 or 18 or 19 or 20 | 9466 |
| 22 | exp Randomized Controlled Trial/ | 522353 |
| 23 | "Randomized Controlled Trial*".tw. | 146201 |
| 24 | "Randomised Controlled Trial*".tw. | 46407 |
| 25 | "Randomized Control Trial*".tw. | 7471 |
| 26 | "Randomised Control Trial*".tw. | 1934 |
| 27 | experiment*.tw. | 2118949 |
| 28 | "clinical trial*".tw. | 389903 |
| 29 | 21 or 22 or 23 or 24 or 25 or 26 or 27 or 28 | 3012422 |
| 30 | 7 and 21 and 29 | 342 |
| 31 | limit 110 to english language | 310 |

Database(s): **CINHAL (EBSCO)** March 30, 2021

| **#** | **Query** | **Results** |
| --- | --- | --- |
| S1 | ((MH "depressive disorder*") OR (AB "depressive disorder*") OR (MH depression) OR (AB depression) OR (MH depressed) OR (AB depressed) OR (MH melanchol*) OR (AB melancholia)) | 160,256 |
| S2 | MH serious games | 483 |
| S3 | TI "serious gam*" OR AB "serious gam*" | 374 |
| S4 | TI "game-based" OR AB "game-based" | 406 |
| S5 | TI "videogame*" OR AB "videogame*" | 307 |
| S6 | TI "video game*" OR AB "video game*" | 2,163 |
| S7 | TI "virtual reality game*" OR AB "virtual reality game*" | 63 |
| S8 | TI "virtual reality-based game*" OR AU "virtual reality-based game*" | 0 |
| S9 | TI "Augmented Reality-based game" OR AB "Augmented Reality-based game" | 0 |
| S10 | TI "Augmented Reality game*" OR AB "Augmented Reality game*" | 21 |
| S11 | TI "gamification" OR AB "gamification" | 353 |
| S12 | TI gamified OR AB gamified | 157 |
| S13 | TI exergam* OR AB exergam* | 359 |
| S14 | TI "Applied game*" OR AB "Applied game*" | 8 |
| S15 | S2 OR S3 OR S4 OR S5 OR S6 OR S7 OR S8 OR S9 OR S10 OR S11 OR S12 OR S13 OR S14 | 4,812 |
| S16 | MH Randomized Controlled Trials | 112,772 |
| S17 | TI Randomized Controlled Trial* OR AB Randomized Controlled Trial* | 93,700 |
| S18 | TI Randomised Controlled Trials OR AB Randomised Controlled Trials | 21,159 |
| S19 | TI "Randomised Control Trial*" OR AB "Randomised Control Trial* | 1,383 |
| S20 | TI "Randomized Control Trial*" OR AB "Randomized Control Trial*" | 3,607 |
| S21 | TI Experiment* OR AB Experiment* | 140,991 |
| S22 | TI "Clinical trial*" OR AB "Clinical trial*" | 112,633 |
| S23 | S16 OR S17 OR S18 OR S19 OR S20 OR S21 OR S22 | 398,482 |
| S24 | S1 AND S15 AND S23 | 89 |

**APA PsycInfo (EBSCO):** March 30, 2021

| **#** | **Query** | **Results** |
| --- | --- | --- |
| S1 | ((MH "depressive disorder*") OR (AB "depressive disorder*") OR (MH depression) OR (AB depression) OR (MH depressed) OR (AB depressed) OR (MH melanchol*) OR (AB melancholia)) | 385,445 |
| S2 | MH serious games | 483 |
| S3 | TI "serious gam*" OR AB "serious gam*" | 374 |
| S4 | TI "game-based" OR AB "game-based" | 406 |
| S5 | TI "videogame*" OR AB "videogame*" | 307 |
| S6 | TI "video game*" OR AB "video game*" | 2,163 |
| S7 | TI "virtual reality game*" OR AB "virtual reality game*" | 63 |
| S8 | TI "virtual reality-based game*" OR AU "virtual reality-based game*" | 0 |
| S9 | TI "Augmented Reality-based game" OR AB "Augmented Reality-based game" | 0 |
| S10 | TI "Augmented Reality game*" OR AB "Augmented Reality game*" | 21 |
| S11 | TI "gamification" OR AB "gamification" | 353 |
| S12 | TI gamified OR AB gamified | 157 |
| S13 | TI exergam* OR AB exergam* | 359 |
| S14 | TI "Applied game*" OR AB "Applied game*" | 8 |
| S15 | S2 OR S3 OR S4 OR S5 OR S6 OR S7 OR S8 OR S9 OR S10 OR S11 OR S12 OR S13 OR S14 | 4,812 |
| S16 | MH Randomized Controlled Trials | 112,772 |
| S17 | TI Randomized Controlled Trial* OR AB Randomized Controlled Trial* | 93,700 |
| S18 | TI Randomised Controlled Trials OR AB Randomised Controlled Trials | 21,159 |
| S19 | TI "Randomised Control Trial*" OR AB "Randomised Control Trial* | 1,383 |
| S20 | TI "Randomized Control Trial*" OR AB "Randomized Control Trial*" | 3,607 |
| S21 | TI Experiment* OR AB Experiment* | 140,991 |
| S22 | TI "Clinical trial*" OR AB "Clinical trial*" | 112,633 |
| S23 | S16 OR S17 OR S18 OR S19 OR S20 OR S21 OR S22 | 496,744 |
| S24 | S1 AND S15 AND S23 | Display |
| S25 | S1 AND S15 AND S23 | 125 |
| S26 | S1 AND S15 AND S23; Narrow by Language: - english | 109 |

| **Database** | **Query** | **Results** |
| --- | --- | --- |
| **Scopus** | ( TITLE-ABS-KEY ("depressive disorder*" OR depression OR depressed OR melanchol* ) ) AND ( TITLE-ABS-KEY ( "serious gam*" OR "game-based" OR "videogame*" OR "video game*" OR "virtual reality game*" OR "virtual reality-based game*" OR "Augmented Reality-based game" OR "Augmented Reality game*" OR "gamification" OR gamified OR exergam* OR "Applied game*" ) ) AND ( TITLE-ABS-KEY ( "randomized controlled trial*" OR "randomised controlled trial*" OR "randomized control trial*" OR "randomised control trial*" OR "clinical trial*" OR experiment* ) ) AND ( LIMIT-TO ( DOCTYPE , "ar" ) OR LIMIT-TO ( DOCTYPE , "cp" ) ) AND ( LIMIT-TO ( EXACTKEYWORD , "Human" ) ) AND ( LIMIT-TO ( LANGUAGE , "English" ) ) | 325 |
| **IEEE Xplore** | ((((Abstract:control trial* OR controlled trial*))) AND ((Abstract: ("depressive disorder*" OR depression OR depressed OR melanchol*) AND ((Abstract:"serious gam*" OR "game-based" OR "video game*" OR "gamification" OR exergam*)) | 2 |
| **ACM Digital library** | [[Abstract: "depressive disorder"] OR [Abstract: depression] OR [Abstract: depressed] OR [Abstract: melanchol*]] AND [[Abstract: "serious gam*"] OR [Abstract: "game-based"] OR [Abstract: "videogame*"] OR [Abstract: "video game*"] OR [Abstract: "virtual reality game*"] OR [Abstract: "virtual reality-based game*"] OR [Abstract: "augmented reality-based game*"] OR [Abstract: "augmented reality game*"] OR [Abstract: "gamification"] OR [Abstract: gamified] OR [Abstract: exergam*] OR [Abstract: "applied game*"]] AND [[All: "randomized controlled trial*"] OR [All: "randomised controlled trial*"] OR [All: "randomized control trial*"] OR [All: "randomised control trial*"] OR [All: "clinical trial*"] OR [All: experiment*]] | 31 |
| **Google Scholar** | ("depressive disorder*" OR depression OR depressed OR melanchol*) AND ("serious gam*" OR "game-based" OR exergam*) AND ("controlled trial*" OR "control trial*") | 100 |
