# Supplementary material for: The Effectiveness of Serious Games for Alleviating Depression: Systematic Review and Meta-analysis
Source: JMIR Serious Games. 2022 Jan 14;10(1):e32331. doi: 10.2196/32331 (PMC8800090; doi:10.2196/32331)
Supplement: Multimedia Appendix 4 [file games_v10i1e32331_app4.docx]

**Appendix 4: Reviewers’ judgements about each “risk of bias” domain for each included study**

1. Kempf K, Martin S. Autonomous exercise game use improves metabolic control and quality of life in type 2 diabetes patients - a randomized controlled trial. BMC endocrine disorders. 2013 Dec 10;13:57. PMID: 24321337. doi: 10.1186/1472-6823-13-57.

2. Meldrum D, Herdman S, Vance R, Murray D, Malone K, Duffy D, et al. Effectiveness of conventional versus virtual reality-based balance exercises in vestibular rehabilitation for unilateral peripheral vestibular loss: results of a randomized controlled trial. Archives of physical medicine and rehabilitation. 2015 Jul;96(7):1319-28.e1. PMID: 25842051. doi: 10.1016/j.apmr.2015.02.032.

3. Rendon AA, Lohman EB, Thorpe D, Johnson EG, Medina E, Bradley B. The effect of virtual reality gaming on dynamic balance in older adults. Age and ageing. 2012 Jul;41(4):549-52. PMID: 22672915. doi: 10.1093/ageing/afs053.

4. Schumacher H, Stüwe S, Kropp P, Diedrich D, Freitag S, Greger N, et al. A prospective, randomized evaluation of the feasibility of exergaming on patients undergoing hematopoietic stem cell transplantation. Bone marrow transplantation. 2018 May;53(5):584-90. PMID: 29335629. doi: 10.1038/s41409-017-0070-8.

5. Jahouh M, González-Bernal JJ, González-Santos J, Fernández-Lázaro D, Soto-Cámara R, Mielgo-Ayuso J. Impact of an Intervention with Wii Video Games on the Autonomy of Activities of Daily Living and Psychological-Cognitive Components in the Institutionalized Elderly. International journal of environmental research and public health. 2021 Feb 7;18(4). PMID: 33562249. doi: 10.3390/ijerph18041570.

6. Andrade A, Cruz WMD, Correia CK, Santos ALG, Bevilacqua GG. Effect of practice exergames on the mood states and self-esteem of elementary school boys and girls during physical education classes: A cluster-randomized controlled natural experiment. PLoS One. 2020;15(6):e0232392. PMID: 32502164. doi: 10.1371/journal.pone.0232392.

7. Ozdogar AT, Ertekin O, Kahraman T, Yigit P, Ozakbas S. Effect of video-based exergaming on arm and cognitive function in persons with multiple sclerosis: A randomized controlled trial. Multiple sclerosis and related disorders. 2020 May;40:101966. PMID: 32045868. doi: 10.1016/j.msard.2020.101966.

8. Song GB, Park EC. Effect of virtual reality games on stroke patients' balance, gait, depression, and interpersonal relationships. Journal of physical therapy science. 2015 Jul;27(7):2057-60. PMID: 26311925. doi: 10.1589/jpts.27.2057.

9. Vieira Á, Melo C, Machado J, Gabriel J. Virtual reality exercise on a home-based phase III cardiac rehabilitation program, effect on executive function, quality of life and depression, anxiety and stress: a randomized controlled trial. Disability and rehabilitation Assistive technology. 2018 Feb;13(2):112-23. PMID: 28285574. doi: 10.1080/17483107.2017.1297858.

10. Shin JH, Bog Park S, Ho Jang S. Effects of game-based virtual reality on health-related quality of life in chronic stroke patients: A randomized, controlled study. Computers in biology and medicine. 2015 Aug;63:92-8. PMID: 26046499. doi: 10.1016/j.compbiomed.2015.03.011.

11. Zhou H, Al-Ali F, Kang GE, Hamad AI, Ibrahim RA, Talal TK, et al. Application of Wearables to Facilitate Virtually Supervised Intradialytic Exercise for Reducing Depression Symptoms. Sensors (Basel, Switzerland). 2020 Mar 12;20(6). PMID: 32178231. doi: 10.3390/s20061571.

12. Fleming T, Dixon R, Frampton C, Merry S. A pragmatic randomized controlled trial of computerized CBT (SPARX) for symptoms of depression among adolescents excluded from mainstream education. Behavioural and cognitive psychotherapy. 2012 Oct;40(5):529-41. PMID: 22137185. doi: 10.1017/s1352465811000695.

13. Merry SN, Stasiak K, Shepherd M, Frampton C, Fleming T, Lucassen MFG. The effectiveness of SPARX, a computerised self help intervention for adolescents seeking help for depression: randomised controlled non-inferiority trial. BMJ : British Medical Journal. 2012;344:e2598. doi: 10.1136/bmj.e2598.

14. Poppelaars M, Tak YR, Lichtwarck-Aschoff A, Engels RC, Lobel A, Merry SN, et al. A randomized controlled trial comparing two cognitive-behavioral programs for adolescent girls with subclinical depression: A school-based program (Op Volle Kracht) and a computerized program (SPARX). Behaviour research and therapy. 2016 May;80:33-42. PMID: 27019280. doi: 10.1016/j.brat.2016.03.005.

15. Perry Y, Werner-Seidler A, Calear A, Mackinnon A, King C, Scott J, et al. Preventing Depression in Final Year Secondary Students: School-Based Randomized Controlled Trial. J Med Internet Res. 2017 Nov 2;19(11):e369. PMID: 29097357. doi: 10.2196/jmir.8241.

16. Cooney P, Jackman C, Coyle D, O'Reilly G. Computerised cognitive-behavioural therapy for adults with intellectual disability: randomised controlled trial. The British journal of psychiatry : the journal of mental science. 2017 Aug;211(2):95-102. PMID: 28596245. doi: 10.1192/bjp.bp.117.198630.

17. Välimäki M, Mishina K, Kaakinen JK, Holm SK, Vahlo J, Kirjonen M, et al. Digital Gaming for Improving the Functioning of People With Traumatic Brain Injury: Randomized Clinical Feasibility Study. J Med Internet Res. 2018 Mar 19;20(3):e77. PMID: 29555622. doi: 10.2196/jmir.7618.

18. Wijnhoven L, Creemers DHM, Vermulst AA, Lindauer RJL, Otten R, Engels R, et al. Effects of the video game 'Mindlight' on anxiety of children with an autism spectrum disorder: A randomized controlled trial. Journal of behavior therapy and experimental psychiatry. 2020 Sep;68:101548. PMID: 32155470. doi: 10.1016/j.jbtep.2020.101548.

19. Donker T, Cornelisz I, van Klaveren C, van Straten A, Carlbring P, Cuijpers P, et al. Effectiveness of Self-guided App-Based Virtual Reality Cognitive Behavior Therapy for Acrophobia: A Randomized Clinical Trial. JAMA psychiatry. 2019 Jul 1;76(7):682-90. PMID: 30892564. doi: 10.1001/jamapsychiatry.2019.0219.

20. Haberkamp A, Walter H, Althaus P, Schmuck M, Rief W, Schmidt F. Testing a gamified Spider App to reduce spider fear and avoidance. Journal of anxiety disorders. 2021 Jan;77:102331. PMID: 33166870. doi: 10.1016/j.janxdis.2020.102331.
