# Supplementary material for: The Effectiveness of Serious Games for Alleviating Depression: Systematic Review and Meta-analysis
Source: JMIR Serious Games. 2022 Jan 14;10(1):e32331. doi: 10.2196/32331 (PMC8800090; doi:10.2196/32331)
Supplement: Multimedia Appendix 5 [file games_v10i1e32331_app5.docx]

**Appendix 5: GRADE Profile for comparison of Serious games to control or conventional exercises for Depression**

| **Certainty assessment** | | | | | | | **№ of patients** | | **Effect** | | **Certainty** |  |
| --- | --- | --- | --- | --- | --- | --- | --- | --- | --- | --- | --- | --- |
| **Participants  (studies) Follow up** | **Study design** | **Risk of bias** | **Inconsistency** | **Indirectness** | **Imprecision** | **Other considerations** | **Intervention** | **Control** | **Relative (95% CI)** | **Absolute (95% CI)** |  |  |
| **Exergames vs. Conventional exercises** | | | | | | | | | | | | |
| 333 (7 RCTs) | randomised trials | very serious ^a^ | very serious ^b^ | not serious | serious ^c,d^ | none | 166 | 167 | - | SMD **0.32 lower** (0.71 lower to 0.08 higher) | ⨁◯◯◯ VERY LOW |  |
| **Exergames vs. Control** | | | | | | | | | | | | |
| 809 (5 RCTs) | randomised trials | very serious ^e^ | very serious ^f^ | not serious | serious ^c,g^ | none | 420 | 389 | - | SMD **0.39 lower** (0.65 lower to 0.12 lower) | ⨁◯◯◯ VERY LOW |  |
| **CBT games vs. Control** | | | | | | | | | | | | |
| 1229 (7 RCTs) | randomised trials | very serious ^h^ | not serious | not serious | serious ^c,i^ | none | 626 | 603 | - | SMD **0.20 lower** (0.34 lower to 0.07 lower) | ⨁◯◯◯ VERY LOW |  |
| **All serious games vs. Control (subgroup analysis)** | | | | | | | | | | | | |
| 2038 (12 RCTs) | randomised trials | very serious ^j^ | serious ^k^ | not serious | Not serious ^l,m^ | none | 1046 | 992 | - | SMD **0.31 lower** (0.46 lower to 0.16 lower) | ⨁◯◯◯ VERY LOW |  |

**CI:** Confidence interval; **SMD:** Standardised mean difference

#### Explanations

a. Evidence was downgraded by 2 levels because the overall risk of bias was rated as high in six studies and there were some concerns in the remaining study due to issues mainly in measurements of the outcome, selection of the reported results, and deviations from intended interventions.

b. Evidence was downgraded by 2 levels as P=0.005 and I square=67%, indicating high heterogeneity.

c. Evidence was downgraded by 1 level because 95% CI crosses one of MID boundaries for this outcome.

d. MID for this outcome, calculated as ± 0.5 times the standardized mean difference (SMD), is ± 0.16

e. Evidence was downgraded by 2 levels because the overall risk of bias was rated as high in three studies and there were some concerns in the remaining two studies due to issues in measurements of the outcome and selection of the reported results.

f. Evidence was downgraded by 2 levels as P=0.003 and I square=68%, indicating high heterogeneity.

g. MID for this outcome, calculated as ± 0.5 times the standardized mean difference (SMD), is ± 0.195.

h. Evidence was downgraded by 2 levels because the overall risk of bias was rated as high in five studies due to issues mainly in measurements of the outcome, selection of the reported results, and deviations from intended interventions.

i. MID for this outcome, calculated as ± 0.5 times the standardized mean difference (SMD), is ± 0.10

j. Evidence was downgraded by 2 levels because the overall risk of bias was rated as high in eight studies due to issues mainly in measurements of the outcome, selection of the reported results, and deviations from intended interventions.

k. Evidence was downgraded by 1 level as P=0.001 and I square=58%, indicating moderate heterogeneity.

l. MID for this outcome, calculated as ± 0.5 times the standardized mean difference (SMD), is ± 0.155

m. Evidence was not downgraded because 95% CI does not cross any of MID boundaries for this outcome.
